# Machine learning and neuromyelitis optica: pioneering precision in disease diagnosiss

**DOI:** 10.1097/MS9.0000000000003796

**Published:** 2025-08-28

**Authors:** Yumna Shariff, Maliha Khalid, Talha Muhammad, Aminath Waafira

**Affiliations:** a Aga Khan University Hospital, Karachi, Pakistan; b Jinnah Sindh Medical University, Karachi, Pakistan; c King Edward Medical University, Lahore, Pakistan; d The Maldives National University, Malé, Maldives

**Keywords:** machine learning, multiple sclerosis, neuromyelitis optica spectrum disorder, radiomics

## Abstract

Neuromyelitis optica spectrum disorder (sNMOSD) poses significant diagnostic challenges due to its clinical and radiologic similarities with multiple sclerosis (MS), despite requiring distinct therapeutic approaches. Early, accurate differentiation is essential to prevent irreversible disability and avoid inappropriate treatment. Machine learning (ML) offers a powerful means to detect subtle imaging differences, such as peri-ependymal lesions and area postrema involvement, often missed in conventional assessment. Recent advances include radiomics-based logistic regression models achieving AUCs above 0.92, compressed 3D convolutional neural networks outperforming traditional models, and transformer-based fusion networks integrating brain and spinal cord MRI with AUCs exceeding 0.93 for NMOSD classification. Incorporating immunological testing, particularly aquaporin-4 (AQP4) antibody status, further improves discrimination between NMOSD, MS, and myelin oligodendrocyte glycoprotein antibody-associated disease (MOGAD). While promising, generalizability is limited by the scarcity of large, multi-center, diverse datasets. Combining advanced ML techniques with robust immunologic profiling offers a path toward precision diagnostics, enabling earlier intervention and improved outcomes for patients with NMOSD.

*To the Editor,*

Neuromyelitis optica spectrum disorder (NMOSD) is particularly challenging to diagnose because its clinical and radiologic characteristics tend to be superimposable with those of multiple sclerosis (MS). Precise differentiation is important, since NMOSD and MS are pathophysiologically distinct conditions that require different treatment strategies. MS is treated mainly by immunomodulatory agents; such therapies are not only ineffective in NMOSD but also potentially disease-enhancing. NMOSD, especially when AQP4-IgG-associated, shows better response to immunosuppressive treatments like rituximab or eculizumab. Hence, early and accurate diagnosis is necessary to avoid irreversible disability.

MRI has subtle changes, such as peri-ependymal lesions in NMOSD, which either extend into the brain parenchyma or line the ventricles and corpus callosum, whereas MS presents with lesions near the third ventricle and hypothalamus^[1]^. Lesions near the aqueduct and fourth ventricle relate to NMOSD pathogenesis via the area postrema, an AQP4-rich, BBB-free zone that may allow NMO-IgG entry into the CNS. Due to the subtlety of the lesion, there is underreporting of the disease^[2]^.

Machine learning helps in identifying even minor changes, and is able to see even what the human eye can miss. A 2021 study extracted 874 radiomic features from quantitative susceptibility mapping (QSM) in deep gray matter. It combined radiomics and demographic data with logistic regression, achieving AUC = 0.927 for discriminating NMOSD from MS^[3]^. Similarly, Wang et al employed a compressed 3D convolutional neural network with two-view blocks and transfer learning, which gave a 75% accuracy, outperforming the conventional 3D CNNs even with limited datasets^[4]^. Another transformer based deep learning algorithm for discriminating demyelinating diseases, used a co-Scale conv-attentional transformer (CoaT) fusion model that was trained with combined brain and spinal cord MRI achieved an overall improved performance, with the AUC of 0.933, 0.942, and 0.803 for MS, AQP4 + NMOSD, and MOGAD, respectively. This exceeded the performance using the brain or spinal cord MRI alone for the identification of the NMOSD complex cases^[5]^.

Although machine learning models exhibit encouraging discrimination between NMOSD and MS, their validity is still constrained by the limited availability of heterogeneous, multi-center datasets. The majority of the data sets are based on homogeneous cohorts drawn from individual institutions, increasing the possibility of diagnostic bias, especially in ethnic groups, different age groups, and seronegative conditions.

There is a need for further advancements, combining immunsological testing with machine learning to revolutionize the diagnosis process. In a recent study, a radiomic nomogram was integrated with AQP4-positive neuromyelitis optica spectrum disorder (NMOSD) to differentiate myelin oligodendrocyte glycoprotein antibody-associated disease (MOGAD) from AQP4-positive NMOSD, yielding an AUC of 0.915 in the original cohort and 0.837 in the validation cohort. Current developments underscore the promise of combining machine learning with aquaporin-4 (AQP4) antibody testing to streamline and optimize the diagnosis of neuromyelitis optica spectrum disorder (NMOSD)^[5]^.

The incorporation of machine learning in the diagnostic pathway is therefore a powerful and precise approach in differentiating neuromyelitis optica spectrum disorder from other demyelinating disorders. These models improve diagnostic accuracy using advanced imaging and immunological markers in complex cases. More research and development of multicenter, diverse datasets will further strengthen the generalizability and clinical applicability of these tools. Our work is in line with the TITAN Guidelines on the need for transparency in AI use in healthcare^[6]^

## Data Availability

No data were generated for this manuscript.

## Peer and provenance statement

This article was not commissioned and was externally peer-reviewed.
